# *Wolbachia*-Induced Cytoplasmic Incompatibility Is Associated with Decreased *Hira* Expression in Male *Drosophila*

**DOI:** 10.1371/journal.pone.0019512

**Published:** 2011-04-29

**Authors:** Ya Zheng, Pan-Pan Ren, Jia-Lin Wang, Yu-Feng Wang

**Affiliations:** Hubei Key Laboratory of Genetic Regulation and Integrative Biology, College of Life Science, Huazhong Normal University, Wuhan, People's Republic of China; Wellcome Trust Centre for Stem Cell Research, United Kingdom

## Abstract

**Background:**

*Wolbachia* are obligate endosymbiotic bacteria that infect numerous species of arthropods and nematodes. *Wolbachia* can induce several reproductive phenotypes in their insect hosts including feminization, male-killing, parthenogenesis and cytoplasmic incompatibility (CI). CI is the most common phenotype and occurs when *Wolbachia*-infected males mate with uninfected females resulting in no or very low numbers of viable offspring. However, matings between males and females infected with the same strain of *Wolbachia* result in viable progeny. Despite substantial scientific effort, the molecular mechanisms underlying CI are currently unknown.

**Methodology/Principal Findings:**

Gene expression studies were undertaken in *Drosophila melanogaster* and *D. simulans* which display differential levels of CI using quantitative RT-PCR. We show that *Hira* expression is correlated with the induction of CI and occurs in a sex-specific manner. *Hira* expression is significantly lower in males which induce strong CI when compared to males inducing no CI or *Wolbachia*-uninfected males. A reduction in *Hira* expression is also observed in 1-day-old males that induce stronger CI compared to 5-day-old males that induce weak or no CI. In addition, *Hira* mutated *D. melanogaster* males mated to uninfected females result in significantly decreased hatch rates comparing with uninfected crosses. Interestingly, *w*Mel-infected females may rescue the hatch rates. An obvious CI phenotype with chromatin bridges are observed in the early embryo resulting from *Hira* mutant fertilization, which strongly mimics the defects associated with CI.

**Conclusions/Significance:**

Our results suggest *Wolbachia*-induced CI in *Drosophila* occurs due to a reduction in *Hira* expression in *Wolbachia*-infected males leading to detrimental effects on sperm fertility resulting in embryo lethality. These results may help determine the underlying mechanism of CI and provide further insight in to the important role *Hira* plays in the interaction of *Wolbachia* and its insect host.

## Introduction

*Wolbachia* are endosymbiotic bacteria that infect many species of arthropods and filarial nematode [1], [2]. The successful spread of *Wolbachia* can be partially attributed to their powerful ability to alter host reproduction by mechanisms such as cytoplasmic incompatibility (CI), parthenogenesis, male killing, and feminization. CI is the most common phenotype, which is expressed as embryonic lethality when *Wolbachia*-infected males mate with uninfected females or with females infected with a different *Wolbachia* strain [3].

Although the molecular mechanism of CI has not been elucidated, several studies suggest that sperm is modified by *Wolbachia* during spermatogenesis. This modification prevents the paternal chromosomes from entering the anaphase of the first division, thus leading to a defect in embryogenesis except that the same *Wolbachia* strain is present in the egg and rescue of CI occurs resulting in hatched embryos [3]–[5]. Studies of *Wolbachia* during spermatogenesis in *Drosophila* have led to a *Wolbachia*-Infected spermatocyte/spermatid hypothesis, which suggests that all CI-expressing strains have *Wolbachia*-infected spermatocytes/spermatids (WISS+cysts) [6]. However, subsequent work in other insect hosts suggests there is little correlation between *Wolbachia* density in spermatocytes/spermatid and the strength of CI. For example, in the parasitic wasp *Nasonia vitripennis*, *Wolbachia* are found only in around 28% of developing sperm, but induce almost complete CI with nearly 100% embryo mortality [7], [8]. This is probably because *Wolbachia* may produce an unknown diffusible CI-inducing factor that can spread from infected to uninfected cells throughout the testis [7]. When the sperm derived from *Wolbachia*-infected males fertilizes uninfected eggs, the first mitotic division in the embryos is severely disrupted. As a result, when the fertilized egg proceeds to anaphase, paternal chromosomes either fail to segregate or appear as extensive chromosome bridging and fragmentation during segregation, indicating damaged or incompletely replicated chromosomes [9], [10]. Tram and Sullivan observed that a delay in nuclear envelope breakdown and activation of cyclin-dependent kinase 1 (cdk1) in the male pronuclei occurred relative to that in the female pronuclei in *Nasonia* [11]. This delay is thought to slow down chromosome condensation in male pronuclei as cdk1 activation is required to drive chromosome condensation [12]. Recently, Landmann *et al.* found that CI delayed deposition of histone H3.3/H4 complex in the male pronucleus, which could be the cause of the chromosome defects present during the first mitotic division in CI embryos [13].

HIR/HIRA, a chaperone of histone H3.3, was first identified in yeast as a negative regulator of histone gene expression [14]. It contains a conserved family of proteins found in various organisms including *Drosophila*, *Xenopus*, mice and human and plays an essential role in development [15]. In *Drosophila*, a point mutation of *Hira* gene (*Hira* ^ssm^, originally called *sésame* gene) causes female sterility. When the eggs laid by homozygous *ssm* females are fertilized,the formation of male pronuclei is arrested in the late chromatin decondensing stage. Therefore the paternal chromatin can not participate in the embryonic development [16]. Further studies have demonstrated that HIRA functions in replication-independent deposition of H3.3–H4 tetramers in the male pronucleus [17]. Loss of function allele (*Hira* ^HR1^) reveals that the HIRA has the only essential role in the assembly of paternal chromatin during male pronucleus formation, since the mutation does not affect the viability of the flies [18].

As both CI embryos and *Hira* mutated flies result in defects in the formation of the male pronucleus, we investigated whether the strength of *Wolbachia*-induced CI is correlated with *Hira* expression level in *Drosophila* flies. Our results show that in both *Drosophila melanogaster* and *Drosophila simulans* males infected by *Wolbachia* strains that induce strong CI, *Hira* expression levels are significantly decreased compared to males exhibiting no CI or *Wolbachia*-uninfected males. In addition, *Hira* expression in 1-day-old *Wolbachia*-infected males inducing strong CI is also significantly reduced relative to 5-day-old *Wolbachia*-infected males exhibiting weak or no CI. Furthermore, we demonstrate that *Hira* mutated male flies mimic the CI phenotype, suggesting that *Wolbachia*-induced CI in *Drosophila* may occur by reducing *Hira* expression in male flies. These results provide an important insight into a novel pathway in which *Wolbachia* interacts with its insect hosts.

## Results

### Reduced *Hira* expression in males expressing strong CI

To test the correlation of CI strength with *Hira* expression level, we initially tested the CI strength in 1-day-old *D. melanogaster* males reared under uncrowded conditions [19]. The results of crossing experiments show that CI is only induced in matings between 1-day-old Dmel *w*Mel males and uninfected Dmel T females (hatch rate of 8.78±1.03). In contrast, no CI is induced by Dmel *w*Au 1-day-old males (hatch rate of 95.55±1.26) (Table 1).

**Table 1:** *D. melanogaster* crosses with different *Wolbachia* strains and host male ages.

Expected CI type	Cross ( male×female)	Egg counted	Egg hatch (%)
**Compatible**	Dmel T (1-day-old)×Dmel T	521	91.70±0.68
	Dmel *w*Mel (1-day-old)×Dmel *w*Mel	551	91.99±0.18
	Dmel *w*Au (1-day-old)×Dmel *w*Au	650	90.04±3.65
	Dmel T (1-day-old)×Dmel *w*Mel	479	91.66±2.92
	Dmel T (1-day-old)×Dmel *w*Au	530	87.02±6.29
	Dmel T (5-day-old)×Dmel *w*Mel	619	92.99±1.36
**Incompatible**	Dmel *w*Mel (1-day-old)×Dmel T	698	8.78±1.03**
	Dmel *w*Au (1-day-old)×Dmel T	737	95.55±1.26
	Dmel *w*Mel (5-day-old)×Dmel T	639	89.51±1.33

Females used in this study were all 3∼4 days old; Egg hatch was shown as mean ± standard error;

**indicates *P*<0.01. The same as in Table 2.

Abbreviations: Dmel *w*Mel, *Drosophila melanogaster* infected with *w*Mel; Dmel *w*Au, *Drosophila melanogaster* infected with *w*Au; Dmel T, *Drosophila melanogaster* treated with tetracycline (without *Wolbachia*).

**Figure 1:**
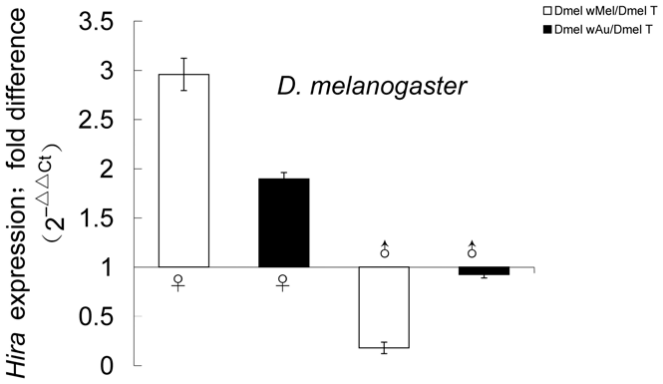
*Hira* gene expression in adult *Drosophila melanogaster* flies. Quantitative RT-PCR was performed on 1-day-old males and 3∼4-day-old females infected with the *w*Mel and *w*Au strains of *Wolbachia* in addition to *Wolbachia*-uninfected flies (Dmel T). “**/**” represented the relative value; bars = standard error; “*” indicated *P*<0.05, “**” indicated *P*<0.01. Abbreviations: Dmel *w*Mel, *Drosophila melanogaster* infected with *w*Mel; Dmel *w*Au, *Drosophila melanogaster* infected with *w*Au; Dmel T, *Drosophila melanogaster* treated with tetracycline (without *Wolbachia*).

To determine whether *Hira* expression is involved in CI level in *D. melanogaster* males, a quantitative RT-PCR assay was performed on 1-day-old male flies. As shown in Figure 1, *Hira* expression was significantly lower in *w*Mel-infected males relative to uninfected males (Dmel *w*Mel/Dmel T: 0.15±0.05) (*P*<0.01). In contrast, *Hira* expression was ∼3 fold higher in *w*Mel-infected females relative to uninfected females (Dmel *w*Mel/Dmel T: 2.96±0.16) (*P*<0.05). Surprisingly, the non CI inducing *w*Au strain did not result in dramatically decreased *Hira* expression in males (Dmel *w*Au/Dmel T: 0.92±0.03). *Hira* expression was 1.90 fold higher in *w*Au-infected females relative to uninfected females (Dmel *w*Au/Dmel T: 1.90±0.07) (*P*<0.05) (Figure 1).

In order to determine if the correlation between CI level and *Hira* expression occurs in additional combinations of *Wolbachia* and their hosts, we examined *Hira* expression in *D. simulans* infected with *w*Ri and *w*Au. Crossing experiments were undertaken with *D. simulans* lines and strong CI was observed to occur in matings between 1-day-old males infected with *w*Ri and Dsim T females (hatch rate of 7.67±2.18). As in *D. melanogaster*, the *w*Au strain did not induce CI in *D. simulans* (hatch rate of 87.62±2.67) (Table 2).

**Table 2:** *D. simulans* crosses with different *Wolbachia* strains and host male ages.

Expected CI type	Cross ( male×female)	Egg counted	Egg hatch (%)
**Compatible**	Dsim T (1-day-old)×Dsim T	355	92.76±3.83
	Dsim *w*Ri (1-day-old)×Dsim *w*Ri	352	91.55±3.08
	Dsim *w*Au (1-day-old)×Dsim *w*Au	301	90.69±3.77
	Dsim T (1-day-old)×Dsim *w*Ri	516	90.43±1.25
	Dsim T (1-day-old)×Dsim *w*Au	393	90.01±1.57
	Dsim T (5-day-old)×Dsim *w*Ri	570	86.16±3.56
**Incompatible**	Dsim *w*Ri (1-day-old)×Dsim T	401	7.67±2.18^**^
	Dsim *w*Au (1-day-old)×Dsim T	347	87.62±2.67
	Dsim *w*Ri (5-day-old)×Dsim T	728	50.89±5.56^**^

Abbreviations: Dsim *w*Ri, *Drosophila simulans* infected with *w*Ri; Dsim *w*Au, *Drosophila simulans* infected with *w*Au; Dsim T, *Drosophila simulans* treated with tetracycline (without *Wolbachia*).

**Figure 2:**
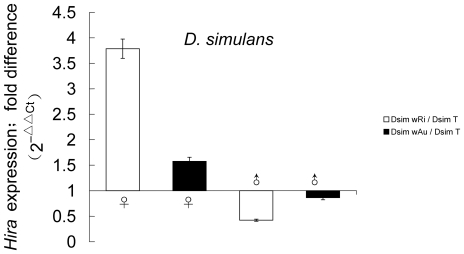
*Hira* gene expression in adult *Drosophila simulans* flies. Quantitative RT-PCR was performed on 1-day-old males and 3∼4-day-old females infected with the *w*Ri and *w*Au strains of *Wolbachia* in addition to *Wolbachia*-uninfected flies (Dsim T). “**/**” represented the relative value; Bars indicated standard error; “*” indicated significant difference (*P*<0.05), “**” indicated extremely significant difference (*P*<0.01). Abbreviations: Dsim *w*Ri, *Drosophila simulans* infected with *w*Ri; Dsim *w*Au, *Drosophila simulans* infected with *w*Au; Dsim T, *Drosophila simulans* treated with tetracycline (without *Wolbachia*).

*Hira* expression in *D. simulans* 1-day-old males was similar to *D. melanogaster* with the CI-inducing *w*Ri strain showing significant down-regulated of *Hira* expression compared to uninfected males (Dsim T) (Dsim *w*Ri/Dsim T: 0.42±0.11) (*P*<0.05) (Figure 2). As expected, there was no significant difference between the non-CI inducing Dsim *w*Au (*Drosophila simulans* infected with *w*Au) and Dsim T males (Dsim *w*Au/Dsim T: 0.86±0.14). For female *D. simulans* flies, *Hira* expression was dramatically higher in *w*Ri-infected females than that in uninfected females (Dsim *w*Ri/Dsim T: 3.79±1.08) (*P*<0.01) (Figure 2). Although females infected with *w*Au also displayed higher *Hira* expression level than Dsim T females (Dsim *w*Au/Dsim T: 1.58±0.15), this difference was not statistically significant (*P*>0.05).

### Reduced *Hira* expression in younger males expressing strong CI

Reynolds and Hoffmann reported that CI levels declined rapidly with increasing of male age in *Drosophila* strains infected by *Wolbachia* [20]. In order to further investigate the correlation of CI intensity with *Hira* expression in *Wolbachia-*infected male flies, we compared *Hira* expression levels between 1-day-old and 5-day-old males of Dmel *w*Mel. As shown in Table 1, matings between *Wolbachia*-uninfected Dmel T females and 1-day-old Dmel *w*Mel males result in significantly lower hatch rates (8.78±1.03) in comparison to that of the 5-day-old Dmel *w*Mel males (89.51±1.33%) (*P*<0.01). However, in the reciprocal crosses between 1-day and 5-day old *Wolbachia*-uninfected males mated with the Dmel *w*Mel females, there were no significant difference in hatch rates (91.66±2.92% and 92.99±1.36%, respectively) (*P*>0.05). This confirmed that in *D. melanogaster*, 1-day-old males induced strong CI, whereas 5-day-old males expressed no CI.

**Figure 3:**
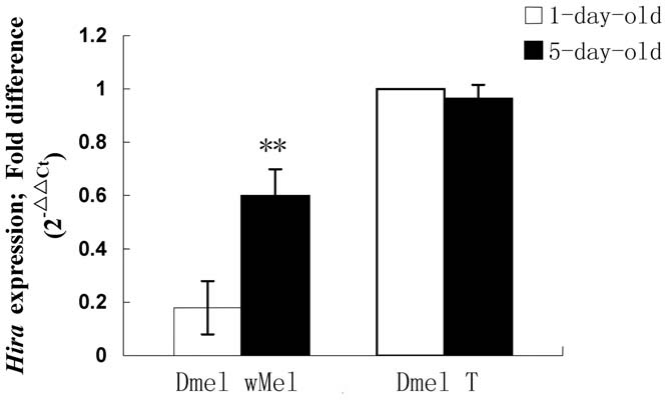
*Hira* gene expression in 1-day and 5-day-old *D. melanogaster* males. Bars indicated standard error; “**” indicated significant difference (*P*<0.01).

*Hira* gene expression, measured by quantitative RT-PCR, increases with age of male Dmel *w*Mel flies (Figure 3). *Hira* expression in 1-day-old males was significantly lower than that in 5-day-old males (*P*<0.01) (Figure 3). However, *Hira* expression was not significantly different between 1-day and 5-day old *Wolbachia*-uninfected males (*P*>0.05) (Figure 3).

**Figure 4:**
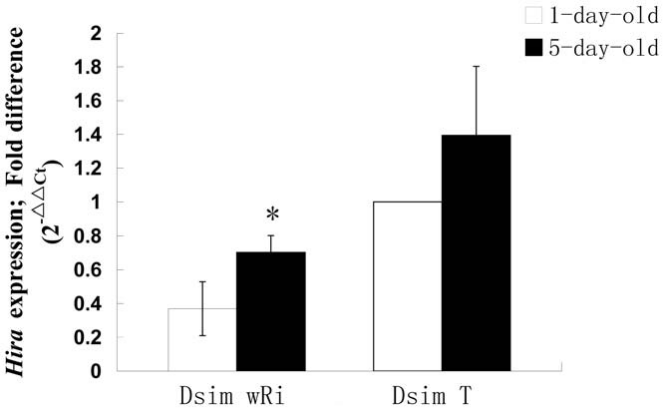
*Hira* gene expression in 1-day and 5-day-old *D. simulans* males. Bars indicated standard error; “*” indicated significant difference (*P*<0.05).

In crosses between Dsim *w*Ri males and uninfected Dsim T females, hatch rates were also correlated with male age. When 5-day-old males were used in the crosses, the hatch rate of the embryos was 50.89±5.56%, which is significantly higher than that in crosses with 1-day-old males (7.67±2.18%) (Table 2). As expected, *Hira* expression in 5-day-old Dsim *w*Ri males was also significantly increased compared to 1-day-old males (*P*<0.05). *Hira* expression between 1-day and 5-day old Dsim T males did not differ significantly (*P*>0.05) (Figure 4) confirming a similar effect in both *D. melanogaster* and *D. simulans*.

### Paternal effects of Hira mutation mimic CI in *D. melanogaster*

**Figure 5:**
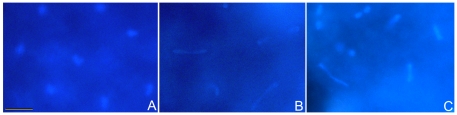
CI expression in early embryos of *D. melanogaster*. (A) Embryos derived from uninfected crosses as control. The nuclei are evenly distributed; (B) Embryos derived from CI crosses with DMel *w*Mel males and DMel T females. Nuclei divisions are asynchronous. Chromatin bridges can be observed. (C) Embryos derived from the crosses of *Hira* ^HR1^ males and DMel T females. Nuclei divisions are asynchronous. Chromatin bridges can be observed. Bars, 10 µm.

To examine the involvement of *Hira* expression in the CI phenotype, the crossing pattern of two *Hira*-mutated *D. melanogaster* lines (*Hira* ^ssm^ and *Hira* ^HR1^) was assessed. Interestingly, we found that both *Hira*-mutated males (1-day-old) mated to *Wolbachia*-uninfected Dmel T females resulted in significantly lower egg hatch rates (72.98±5.10%, 74.34±4.03%, respectively) relative to Dmel T males (92.44±0.77%) (*P*<0.05) (Table 3). However, the crosses between *Hira*-mutated males and *w*Mel-infected females resulted in no significant differences of hatch rate comparing with uninfected crosses (86.81±4.37%, 89.38.34±8.06%, respectively) (Table 3). In the early embryos derived from the crosses of *Hira*-mutant males with Dmel T females, the asynchronous cleavage and chromatin bridges were observed (Figure 5, C) which is similar to the CI phenotype in *D. melanogaster* (Figure 5, B), while in the embryos derived from the uninfected flies the nuclear division was synchronous and the nuclei were evenly distributed (Figure 5, A). This suggests that the mutation of *Hira* in males may mimic the CI phenotype induced by *Wolbachia*.

**Table 3:** Effect of *Hira*-mutant males (1-day-old) on egg hatch rates and progeny sex ratio.

Expected CI type	Cross ( male×female)	Egg hatch (%)	Sex ratio (F∶ M)	Total progeny
**Compatible**	Dmel T×Dmel T	92.44±0.77	1.03±0.20	320
	*Hira* ^ssm^×Dmel *w*Mel	86.81±4.37	1.04±0.17	334
	*Hira* ^HR1^×Dmel *w*Mel	89.38±8.06	1.17±0.26	219
**Reciprocal**	*Hira* ^ssm^×Dmel T	72.98±5.10*	0.68±0.08*	97
	*Hira* ^HR1^×Dmel T	74.34±4.03*	0.62±0.05*	227

Egg hatch and the ratio of female to male were shown as average ± standard error;

*indicates *P*<0.05.

Considering that *Hira* is on the X chromosome, we also examined the offspring sex ratio to see whether *Hira* mutation impacts sperm quantity. As shown in Table 3, mating with both *Hira*-mutant males resulted in significantly less female progeny in the next generation. In the progeny, the ratio of females to males derived from the crosses with *Hira* ^ssm^ and *Hira* ^HR1^ were 0.68 (±0.08) and 0.60 (±0.11), respectively. These sex ratios are significantly lower than those result from crosses with Dmel T males (*P*<0.05), where the ratio was 0.98 (±0.10) (Table 3). These results suggest that many female progeny mortality was associated with *Hira* mutations on one X chromosome. Therefore we conclude that *Hira* mutation has negative effects on sperm quantity. In addition, the presence of the *w*Mel strain of *Wolbachia* in females restored the sex ratio as shown by a sex ratio of ∼1 when *Hira*-mutated males were crossed with Dmel *w*Mel females (Table 3).

## Discussion

Previous studies both *in vitro* and *in vivo* have shown that *Wolbachia* infection may affect the expression of various host genes, including those associated with immunity, fertilization, and development [21]–[23]. For example, *w*MelPop strain of *Wolbachia* has been shown to be capable of inducing immune upregulation in *Anopheles gambiae* and *Aedes aegypti* mosquitoes [23], [24]. Even in a cell line naturally infected with *Wolbachia*, the expression of antioxidant proteins is also upregulated [22]. However, the association of CI strength induced by *Wolbachia* with the gene expression level in hosts is unknown. Here, we used two lines from both *D. melanogaster* and *D. simulans* infected with different *Wolbachia* strains to determine if *Hira* gene expression is correlated with CI. Our results demonstrate that for both Dmel *w*Mel and Dsim *w*Ri 1-day-old males, which express strong CI, the *Hira* expression levels are significantly decreased compared to *Wolbachia*-uninfected and *w*Au-infected males which induce either weak or no CI. Furthermore, increasing male age was correlated with increasing hatch rates (Tables 1, 2). Correspondingly, *Hira* expression was significantly lower in younger males (Figures 3, 4), suggesting that down regulation of *Hira* expression in male *Drosophila* might be causally linked to the CI strength.

The molecular mechanisms of abnormal embryo development in CI crosses are not fully known. Genetic and cellular evidences indicate that in CI embryos, the paternal chromosomes are improperly condensed when aligned at the metaphase plate of the first mitotic division after fertilization [9], [10], [25]. This could be attributed to the modification in the sperm of *Wolbachia*-infected males. Riparbelli *et al.* have described some malformations, including abnormal mitochondria and axoneme in the sperm developed within the infected testes [7]. Additionally, the amount of sperm produced by *Wolbachia*-infected *D. simulans* males is about 40% of that in uninfected males, especially in young males expressing strong CI, indicating that *Wolbachia* could affect male fertility through multiple ways [26]. Studies of spermatogenesis in the parasitic wasp *Nasonia vitripennis* and the beetle *Chelymorpha alternans* revealed that *Wolbachia* can modify sperm despite not being present in developing sperm, suggesting that *Wolbachia* might alter expression and synthesis of gene products in the host, thus changing the products exported to the developing spermatids [8]. In this study, we show that *Wolbachia* strains that induce strong CI (*w*Mel and *w*Ri) significantly decrease *Hira* expression in male *Drosophila* flies, which may impair the sperm function at fertilization and result in the CI phenotype.

Chromatin remodeling is extremely significant in the late stage of spermatogenesis due to the necessity of histone replacement by male-specific transition protein (TP) and later by small arginine-rich proteins named protamines, ensuring the compacted sperm head configuration formation and promoting sperm getting into the female reproductive tract for fertilization [27]. It is known that HIRA is a chaperone of histone variant H3.3 and is involved in a DNA replication-independent pathway of nucleosome assembly [28]. In *Drosophila*, H3.3 is incorporated in specific regions in the early stages of spermatogenesis and then mostly disappears in condensed spermatid nuclei just before protamine deposition [18], [29]. H3.3 could play a role in spermatogenesis. Indeed, male mice carrying an impaired *H3.3A* gene have reduced fertility [30]. Considering that the process of spermatid differentiation is independent of DNA synthesis, the histone H3.3 chaperone HIRA might be involved in this process. In this study, we have shown that *Hira* mutations in young male *Drosophila* flies results in a significantly reduced hatch rates (Table 3) comparing with the uninfected flies, suggesting that HIRA could have an effect on male fertility by acting as a chaperone of H3.3. This is in contrast with the observations by Bonnefoy *et al.* [18], where they showed that *Hira* mutation had no effects on viability and male fertility. The contrasting results may be due to the fact that *Hira* mutated males do not completely lose their fertility but produce significantly less progeny.

Following fertilization, the paternal chromosomes are abnormally condensed during the first zygotic division in the embryos derived from CI crosses [4], [5]. Tram and Sullivan found that in CI crosses, the nuclear envelope breakdown and Cdk1 activation are delayed in the male pronucleus relative to those in the female pronucleus [11]. Recent studies revealed a delay in loading H3.3 onto the paternal chromosomes, possibly causing disruption of replication in the male pronucleus of CI embryos [13]. As the chaperone of H3.3, HIRA has been demonstrated to be essential in sperm chromatin remodeling and specifically in assembling H3.3 containing nucleosomes during the formation of male pronucleus. Female flies homozygous for the null allele of *Hira* are sterile due to a defect in incorporated sperm nucleus decondensation [17], [18]. In this regard, HIRA could be involved in the abnormal deposition of H3.3 to the paternal chromosome in CI embryos. It is possible that the low level of *Hira* in young *Wolbachia*-infected males results in a structural malformation of sperm nucleus. When this sperm fertilize an uninfected egg, HIRA chaperoned maternal H3.3 may not be deposited promptly on the male nucleus during sperm chromatin remodeling. In this study, we have shown that in *Wolbachia*-infected female flies, *Hira* expression is dramatically increased in both Dmel *w*Mel and Dsim *w*Ri (Figures 1, 2). High level of HIRA in the females might be able to compensate for the shortage in sperm nucleus resulted from *Wolbachia* infection, thus speed up the deposition of H3.3 in paternal chromosomes and rescue this defect at fertilization. Several models have been described to explain the rescue mechanism including the hypothesis that *Wolbachia* removes an essential component from the sperm nucleus and the same strain of *Wolbachia* in the egg restores this critical factor allowing embryogenesis to proceed normally. This “titration-restitution” model [31] can explain why CI induction can be suppressed when infected males mate with females infected with the same strain of *Wolbachia* [2]. We have shown here that *Hira*-mutants in young males lead to a higher paternal-effect embryonic lethality with a phenotype of chromatin bridges. Interestingly the egg with *Wolbachia* may rescue the defects and result in high hatch rates. These mimic the CI phenotype induced by *Wolbachia*. Furthermore, it is the sperm- carrying X chromosome in *Hira*-mutants that leads to embryonic lethality, since there are significantly less females produced from these crosses (Table 3). Taken together, our results suggest that *Wolbachia* may induce CI by regulating the expression of some key factors, such as reducing *Hira* expression in males, which may influence sperm fertility and cause CI phenotype. For the compatible crosses of *Wolbachia*-infected females with uninfected males, it is possible that higher level of *Hira* in females has no effects on the embryogenesis. For bidirectional incompatibility, it is likely that different *Wolbachia* strains differentially impact *Hira* expression so that females infected with one *Wolbachia* strain can not rescue the deficiency in the sperm caused by another *Wolbachia* strain.

## Materials and Methods

### Fly lines

Fly lines were kept on a standard corn diet at a constant temperature of 25°C, with 8L ∶ 16D (light ∶ dark) cycle and were reared under non-crowded condition (200±10 eggs per 50 ml vial of media in 150 ml conical flask) [19]. The following *Drosophila* strains were used in the study: *Wolbachia*-infected Dmel *w*Mel (*D. melanogaster* Brisbane nuclear background with introgressed *w*Mel from YW), Dmel *w*Au (*D. melanogaster* Brisbane nuclear background with injected *w*Au), Dsim *w*Ri (*D. simulans* naturally infected with *w*Ri), and Dsim *w*Au (*D. simulans* Coffs Habour). Cured Dmel *w*Mel and Dsim *w*Ri were subsequently generated by tetracycline treatment following established protocols [32] and designated Dmel T and Dsim T, respectively. *D. melanogaster* of *Hira* ^ssm^ (a point mutant of *Hira*) [17] and *Hira* ^HR1^ (a loss of function *Hira* allele) [18] were provided kindly by Dr. Loppin B. at the Université Claude Bernard Lyon I, France. These two *Hira* mutated lines were confirmed to be *Wolbachia*-free by PCR (data not shown).

### CI Assays

CI tests were performed as previously described by Yamada *et al.* [19]. All the crossing schemes including expected compatible and incompatible crosses in this study are shown in Tables 1, 2, and 3. In all crosses, adult virgins were collected and crosses were undertaken with 30 females (3–4 days old) and 20 males (either 1-day or 5-day-old) at 25°C in bottles upturned on agar/grape juice plastic Petri dishes (ca.4cm^2^). After mating for around 10 h, all males were removed from the bottle to avoid diminishing CI effects with increasing male age. Eggs were then collected for 6–8 h and incubated at 25°C and 45–70% humidity for 48 h. Hatch rates were determined by counting the number of hatched eggs to total eggs.

### Quantitative RT-PCR Assay

Quantitative reverse transcription PCR (qRT-PCR) was performed to determine the relative *Hira* gene expression level in different fly lines. Total RNA was extracted from adults (males or females) using Trizol (Invitrogen). DNA contamination was removed with RQ1 DNase (Promega). The first-strand cDNA was synthesized from 2 µg of total RNA using reverse transcriptase (RT) (Promega) and oligo dT15 primer (Takara) at 42°C for 1.5 h. Special primers were designed based on flybase for *Hira* and the reference gene *rp49* as following: *HiraF* (5′-ATGCGGCTCCTTAAGCCGGC-3′) and *HiraR* (5′-ATCTTCGGAACATCCGCATCG-3′); *rp49F* (5′-CGGTTACGGATCGAACAAGC-3′) and *rp49R* (5′-CTTGCGCTTCTTGGAGGAGA-3′). qPCR was performed using a Miniopticon system (BioRad) with a Platinum SYBR Green qPCR superMix (Takara). The reaction volume was 20 µl, containing 10 µl SYBR Premix Ex Taq (2×), 0.15 µl of forward and reverse primer (20 µM), respectively, 7.7 µl ddH_2_O and 2 µl of cDNA template diluted by 10-fold. The qPCR procedure was consisted of 95°C for 2 min, followed by 95°C for 10 s, 61°C for 15 s and 72°C for 10 s per cycle for 40 cycles, then a melting curve analysis was carried out by a slow increase (0.2°C/s) from 55°C to 98°C, in purpose of examining if there were primer-dimers or nonspecific amplification. The relative expression ratio of *Hira* gene for samples A to B was calibrated against *rp49* gene using the 2^−ΔΔCT^ calculation method: ΔΔCT = (CT_Hira_−CT_rp49_) _sampleA_−(CT_Hira_−CT_rp49_) _sampleB_.

### Immunofluorescence

Embryos were collected every 15 minutes and immersed in a 50% bleach solution for 2–3 mins to remove the chorion. Next they were washed in embryo wash buffer (0.7% NaCl, 0.05% Triton X-100) and fixed by vigorous shaking in a 1∶1 heptane/methanol mix for 30s. Embryos were washed three times with methanol and stored in methanol at −20°C until use. Embryos were washed 5 min in −20°C acetone and then three times (10 min each) in TBST (50 mM Tris–HCl, pH 7.4, 50 mM NaCl, 0.02% sodium azide, 0.1% Triton X-100). The samples were stained with DAPI (Beyotime, China) solution for 15 min at room temperature, then washed using TBST for 10 min. Embryos were mounted in the mounting medium (10% PBS, 90% glycerol). The slides were observed and photographed using a Leica DM 4000B fluorescence microscope (Leica, Germany).

### Statistical Analysis

Results are presented as means ± SE (n = 3). Differences among means were analyzed by one-way analysis of variance (one-way ANOVA). Differences were regarded as statistically significant when *P*<0.05.
